# Evaluation of riparian condition of Songhua River by integration of remote sensing and field measurements

**DOI:** 10.1038/s41598-017-02772-3

**Published:** 2017-05-31

**Authors:** Bolin Fu, Ying Li, Yeqiao Wang, Anthony Campbell, Bai Zhang, Shubai Yin, Honglei Zhu, Zefeng Xing, Xiaomin Jin

**Affiliations:** 1 Northeast Institute of Geography and Agroecology, Chinese Academy of Sciences, 4888 Shengbei Street, Changchun, Jilin 130102 P. R. China; 2 University of Chinese Academy of Sciences, Beijing, 100049 P. R. China; 3 Department of Natural Resources Science, University of Rhode Island, Kingston, RI 02881 USA; 4 Tangshan Normal University, 156 Jianshe Street, Tangshan, 063000 P. R. China; 5 Henan Normal University, 46 Jianshe Street, Henan, 453007 P. R. China; 6 Institute of Agricultural Resources and Regional Planning, Chinese Academy of Agricultural Sciences, 12 Zhongguancun Southen Street, Haidian District, Beijing 100081 P. R. China

## Abstract

Riparian zone is crucial to the health of streams and their surrounding environment. Evaluation of riparian condition is essential to achieve and maintain good stream health, as well as to sustain ecological functions that riparian areas provide. This manuscript is aimed to evaluate riparian conditions of Songhua River, the fifth longest river in China, using physical structural integrality (PSI) values derived from remote sensing and validated by field measurements. The variation and clusters of PSI values were discriminated by the spatial statistics to quantify variation of riparian condition in each measurement section. Evaluation results derived from 13 measurement sections indicated that over 60% of the riparian zones have been disturbed by human activities. Analysis of land use patterns of riparian zone in the cold and hot spots found that land-use patterns had an important effect on riparian condition. The build-up and farmland areas had been the main human disturbances to the riparian condition, which were increased from 1976 to 2013. The low-low clusters (low PSI values with low neighbors) of PSI values can be implemented to identify the vulnerability of the riparian zone.

## Introduction

Riparian zones are narrow strips of land located along the banks of rivers, streams, and water networks. Riparian zones are widely acknowledged as an ecological transition zone of material and energy exchange between terrestrial and aquatic ecosystems^1, 2^. Riparian zones can provide a range of ecosystem functions and services, e.g., bank stabilization and protection, water purification, reservoirs of biodiversity, wetland products, as well as recreation and tourism^3–5^. Riparian zones are also a focus of human activities, such as urban expansion, agriculture, mining, grazing, erosion, and point and non-point source pollutions^6–8^. It is essential that riparian zones are managed appropriately to avoid degradation and damage that have become increasingly evident^9–13^.

The physical structural integrality (PSI) characterizes riparian ecological condition using indicators, e.g., bank condition, riparian vegetation condition and human intervention. Methodologies for assessing PSI of riparian zones have been developed to provide different evaluating indicators^9, 10, 14–17^. Most of the methods are based on expert knowledge in selection of measuring sections and monitoring sites, then evaluating the PSI through field measurements. However, those methods are challenged in assessing long stretches of riparian zone, in particular vast regions and remote locations. The evaluating methods have usually been concentrated on field measurements of a few hundred meters, which could be very laborious or even unpractical when attempting to evaluate an entire catchment or a long river corridor^18^. In addition, selection of measuring sections and sites might not be able to take into account simultaneously the representativeness, accessibility and security, which made it difficult to fully characterize a riparian zone with site-based field data alone. Remote sensing techniques have been utilized to map indicators of riparian condition because of advantages in spatial extensiveness, non-invasiveness, and repetitive capability^19–22^. Those studies demonstrated that indicators such as streambed width, riparian zone width, riparian vegetation and bank stability were important and feasible to extract from remote sensing data for condition assessment of riparian zones. However, evaluate riparian conditions using those indicators still remain to be examined.

This study evaluated the riparian condition of the Songhua River across the Northeastern Plain of China, using a series of indicators developed from 2,081 basic evaluation units (BEUs). The specific objectives of this paper are to: (1) demonstrate the feasibility of the multi-metric approach through comparisons with field measurements; (2) discriminate the variation and clusters of riparian condition to identify the vulnerability and stability of the riparian zones; and (3) explore the change of landscape of the riparian zone using the land use data from 1976 to 2013 to understand the effects of human impacts on riparian conditions.

## Materials and Methods

### Study area

**Figure 1:**
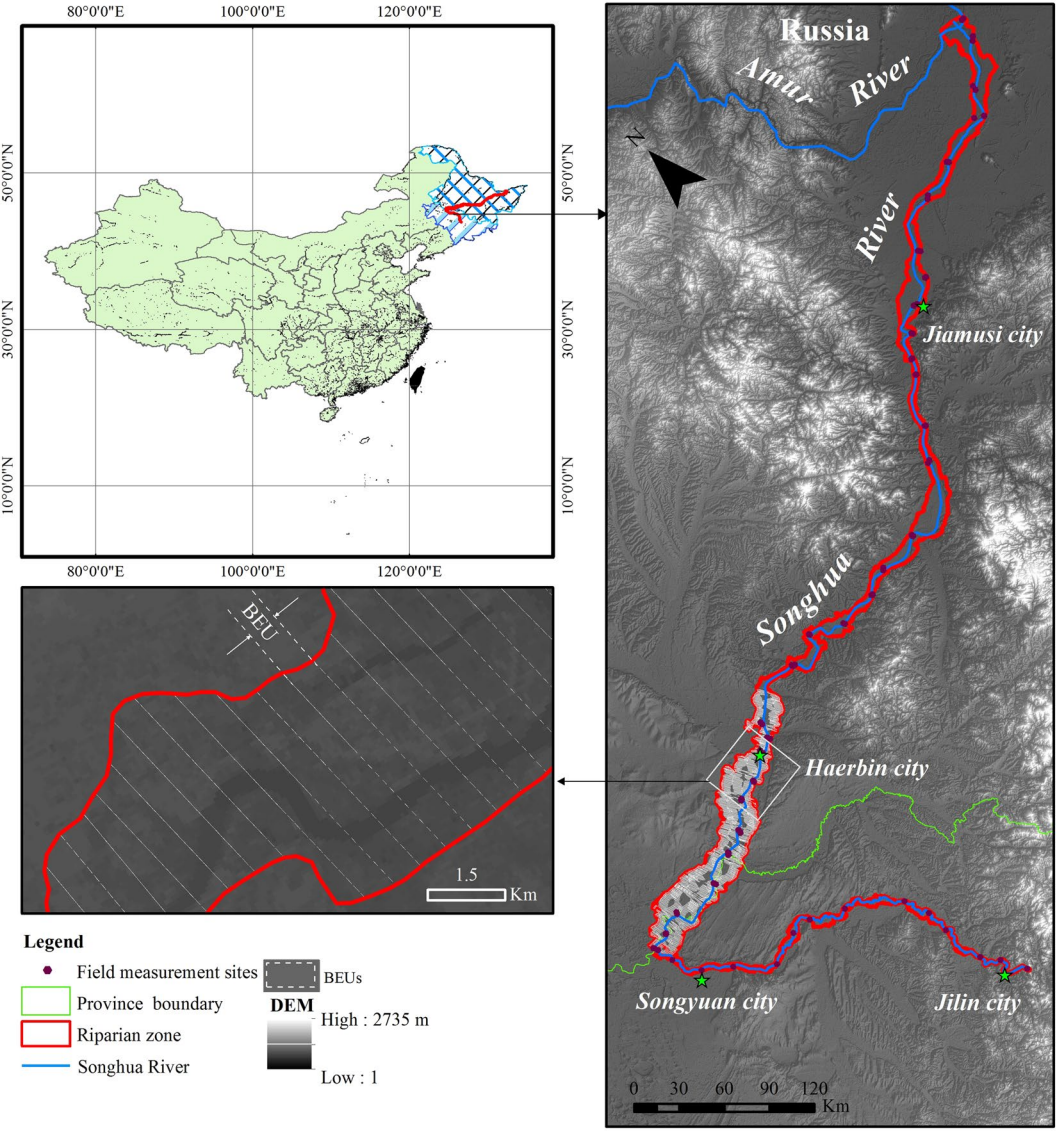
This figure illustrates the study area and field measurement sites in the Northeast China. The riparian zone was divided into 13 measurement sections, and each measurement section was partitioned into basic evaluation units (BEUs).The base map (right) is the Shuttle Radar Topography Mission 1 arc-second global DEM (U.S. Geological Survey, National Geospatial-Intelligence Agency and National Aeronautics and Space Administration, 2000, Shuttle Radar Topography Mission 1 Arc-Second Global, Sioux Falls, South Dakota.: U.S. Geological Survey (USGS) Earth Resources Observation and Science (EROS) Center, https://lta.cr.usgs.gov/SRTM1Arc). ArcGIS 10.3 software (http://www.esri.com/software/arcgis) was used to develop the map.

Songhua River is the largest tributary of the Heilongjiang (Amur River) in the Northeast China. With the length of 1,897 kilometers long and the drainage area of 545,600 square kilometers, Songhua is ranked the fifth longest river in China. It is originated from Changbai Mountain, passing through the Northeastern Plain, and injected into Amur River at about 48° north latitude. This study focused on the 1,679 kilometers riparian zone of Songhua River started from the Fengman hydrologic dam passing through major cities such as Jilin, Harbin, and Jiamusi, and ended at the Amur River, the border river between China and Russia (Fig. 1). The elevation of the river basin is between 54 to 2,735 meters above mean sea level. The basin has temperate continental climate with four clearly distinct seasons. The mean annual rainfall in the area is about 550–800 millimeter. The river reaches its maximum flow in summer and is frozen from November to April next year.

Songhua River basin is a major agricultural center and commodity grain production base of China. Large scale operations of state farms were established after the establishment of the People’s Republic of China in 1949 and land reclamation projects begun since. Songhua River is an important transportation artery for agricultural products of the region. The river flows through major port cities and serves as the source of drinking water for millions of people. The ecological functions and integrality of riparian zones of the Songhua River have experienced much degradation under the influence of anthropogenic activities such as overgrazing, construction of transportation infrastructure, urban sprawl, sand mining, tourism development, reclaimed wetland and other human activities.

### Land cover data and ancillary data

**Figure 2:**
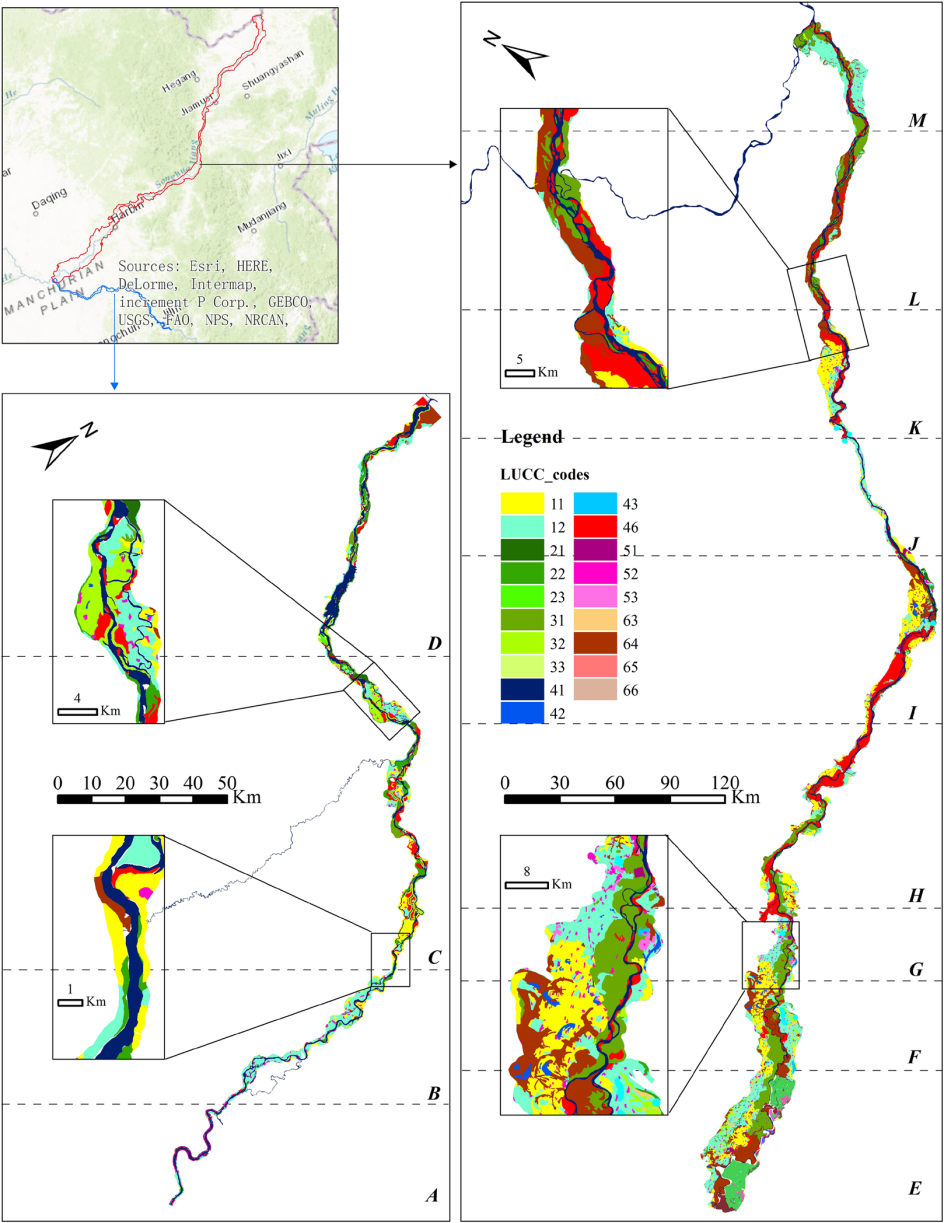
This figure illustrates the land-use data at 1:100,000 scale for riparian zone in 2012. The LUCC_codes represent different land-use types, i.e., 11–12: paddy field, glebe field; 21–23: arboreal forest, sparse woodland and shrub woodland; 31–33: high-, mid- and low-cover grassland; 41–43: rivers, lakes and reservoirs; 46: beach; 51–53: residential, rural and transportation; 63: saline-alkali soil land; 64: wetland; 65–66: Barren land. ArcGIS 10.3 software (http://www.esri.com/software/arcgis) was used to process and map the data by first author (B. F.). The locus map is the World Topographic Map basemap within ArcGIS 10.3 software. The map is credited to: Esri, HERE, DeLorme, Intermap, increment P Corp., GEBCO, USGS, FAO, NPS, NRCAN, GeoBase, IGN, Kadaster NL, Ordnance Survey, Esri Japan, METI, Esri China (Hong Kong), swisstopo, MapmyIndia, © OpenStreetMap contributors, and the GIS User Community.

This study adopted the land-use data at 1:100,000 scale (Fig. 2) for 1976, 1986, 1995, 2000, 2005 and 2013 from the Resources and Environment Science Data Center, Chinese Academy of Sciences. The land-use datasets included information of *forest*, *grassland*, *farmland*, *wetland*, *water body*, *barren land* and *build-up* (Table 1). The land use dataset of 2013 and 2015 derived from updating the land use map of 2005 with interpretation keys from field measurement sites using Landsat 8 Operational Land Imager (OLI) images acquired in 2013 and 2015, respectively. Table 2 describes selected multi-spectral OLI images that covered the study area with 30 m spatial resolution.

**Table 1:** The categories of 1:100,000 land use data in riparian zone.

Categories	Sub-categories
Forest	arboreal forest, sparse woodland and shrub woodland
Grassland	high-, mid- and low-cover grassland
Farmland	paddy field, glebe field
Wetland	marsh, riverine wetland
Water body	rivers, reservoirs fishery and lakes
Barren land	lands unused or difficult for using, saline-alkaline land
Build-up	industrial and commercial, residential, transportation

**Table 2:** Multi-spectral Landsat 8 OLI images.

Years	Path/Row	Acquisition Date	Sensor
2013	118/29	17 Sep 2014	OLI
	119/29	24 Sep 2014	
2015	115/27	15 Sep 2015	OLI
	116/27	19 Sep 2014	
	116/28	06 Sep 2015	
	117/28	13 Sep 2015	
	118/28	04 Sep 2015	
	119/28	24 Sep 2014	

**Figure 3:**
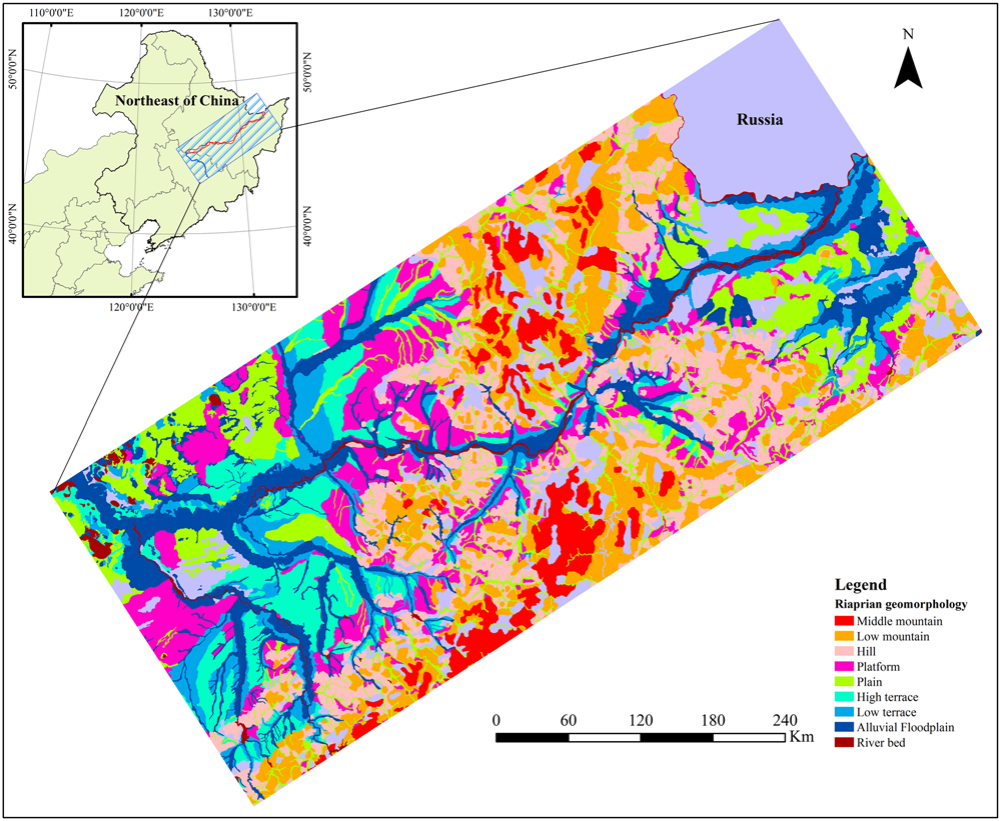
This figure illustrates an example of 1:500,000 geomorphic map for the riparian zone. The categories include Middle mountain, Low mountain, Hill, Platform, Plain, High terrace, Low terrace, Alluvial Floodplain and River bed. ArcGIS 10.3 software (http://www.esri.com/software/arcgis) was used to process and map the data by first author (B. F.).

Other datasets adopted including 1:500,000 geomorphic map (Fig. 3) and 1:100,000 topographic map developed by the Institute of Geographic Sciences and Natural Resources Research, Chinese Academy of Sciences, and Shuttle Radar Topographic Mission (SRTM) generated Digital Elevation Model (DEM) data at 30 m spatial resolution. Referencing the 1:100,000 topographic map and 1:500,000 geomorphic map, the elevation range of the riparian zone was between 115 and 370 meters.

### Field data collection

**Figure 4:**
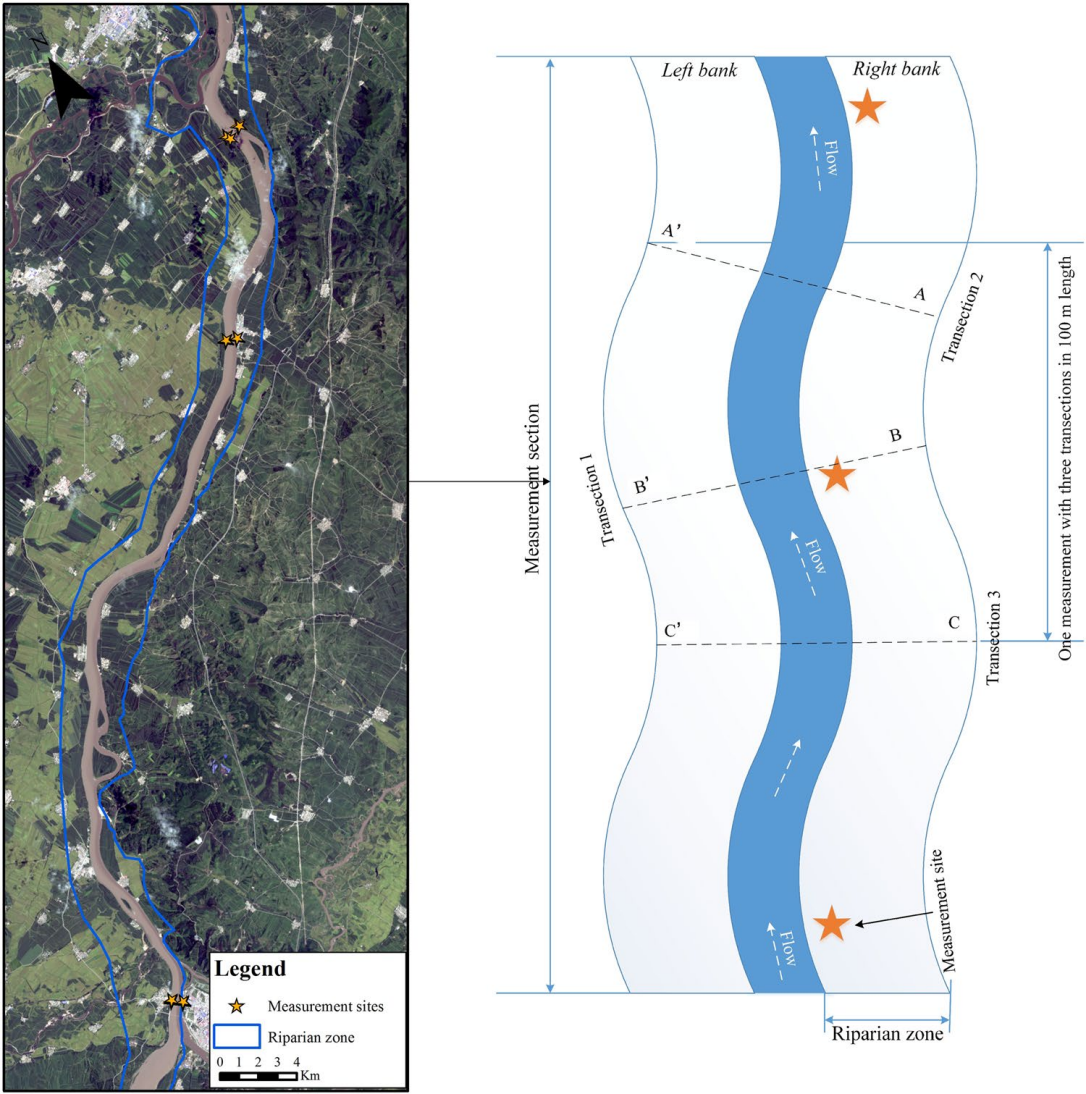
Landsat 8 OLI imagery (left) acquired in September 06, 2015 (U.S. Geological Survey (USGS) Earth Resources Observation and Science (EROS) Center, 2015, Landsat_8 – Path 116 Row: 28 for Scene: LC81160282015249LGN00, Sioux Falls, South Dakota.: U.S. Geological Survey (USGS) Earth Resources Observation and Science (EROS) Center, https://lta.cr.usgs.gov/L8) illustrated example locations of the measurement sites and the layout of field measurements (right). One measurement section had 3–4 measurement sites, one measurement site had three transections with 30 m width × 50 m length measurement area on both side of banks. ArcGIS 10.3 software (http://www.esri.com/software/arcgis) was used to process and map the data by first author (B. F.).

The studied riparian zone was divided into 13 measurement sections according to the Chinese Ministry of Water Resources technical protocol^11^. Within each section three or four measurement sites were chosen, and each site consisted of three transections (Fig. 4). Each transection was located in an area with a consistent management regime and with similar vegetation and stream characteristics. The entire riparian zone consisted of 45 measurement sites (Fig. 1) and 135 transections. Each transection laid two 30 m width × 50 m length measurement area on both side of banks. The positions of measurement sites and transections were guided by Global Positioning System (GPS). The sub-indicators of field-based PSI include *bank slope* and *slope length* of river bank, *Canopy cover*, *bank sediment size* (clay, sand, gravel, cobbles or bedrock), *bank erosion* and *anthropogenic activities* (Table 3).

**Table 3:** The field-based indicators and weights of PSI.

Indicators		Sub-indicators
Riparian Stability (RST) (0.5)	Bank stability (BKS) (0.25)	Bank Slope (BS) (0.2)
		Slope height (SH) (0.2)
		Vegetation percent cover (VPC) (0.2)
		Bank sediment size (BSS) (0.2)
		Erosion status (ES) (0.2)
	Vegetation Coverage (BVC) (0.5)	Canopy cover
	Human disturbance (RD) (0.25)	Human built structures, agriculture, transportations, sand mining and other activities
River connectivity (RC) (0.25)		Human-built instream structures, including bridges, dams, culverts
Natural wetland conservation (NWC) (0.25)		Wetland area in the evaluating year (A_C_) and historical year (1976)(A_R_)

The *bank slope* and *slope length* were directly measured by a laser rangefinder, and used to calculate *slope height*. *Canopy cover* was measured by 1 × 1 m quadrat within the 30 × 50 m evaluation area. The 1 × 1 m vegetation quadrat was photographed with a camera mounted on a vertical shooting platform. These photos were subsequently processed to calculate vegetation coverage in every quadrat with the binary segmentation method by image-processing, and then calculated all *Canopy cover* in the evaluation area. *Anthropogenic activities*, *bank sediment size*, *erosion* and *river connectivity* were recorded by field photos. All field measurements were executed during the normal river flow period, in Septembers of 2013 and 2015, respectively.

### Description of BEUs

The BEUs is defined as the basic evaluation units with various sizes in the measurement sections of the riparian zone (Fig. 1). To obtain the BEUs, the riparian zone was retrieved as a single featured GIS polygon using the 1:500,000 geomorphic map (Fig. 3). The river centerline was extracted from the river mouth to the source for the main channel based on the 1:100,000 topographic map and the DEM. The 600 m length was adopted following the Chinese Ministry of Water Resources technical protocol^11^ as a splitting criterion to divide the river centerline. Then, the polygon of the riparian zone was separated using splitting lines perpendicular to the river centerline. This process generated 2,081 BEUs in the study area. The minimum, average and maximum size of the BEUs were 0.28 km^2^, 2.89 km^2^ and 19.81 km^2^, respectively. All indicators and sub-indicators were calculated and scored based on the grid of BEUs.

### PSI Indicators derived from remote sensing data

Adapting field-based indicators to remote sensing has been done through a combination of direct conversion and substitution (Table 4). *Bank slope* was calculated by DEM. *Vegetation percent cover* (VPC) and *human disturbance* (RD) were calculated by the Equation (1) and (2) using the updated 1:100,000 land use data. The width of riparian zone is affected synthetically by bank sediment size, flow erosion and deposition^23^. Previous studies utilized *water level width* and *riparian zone area* as sub-indicators to assess *bank stability* ^24–26^. In addition, these sub-indicators could be accurately calculated by remote sensing data. In this paper, the *area of riparian zone* was calculated based on the 1:500,000 geomorphic map (Fig. 3) and 1:100,000 topographic map. The *water level width* of transection was extracted using OLI images. The two sub-indicators were normalized for converting the value to 0–100 (Equation (3)). *Natural wetland conservation* (NWC) was calculated using the 1976, 2013 and 2015 1:100,000 land-use data (Equation (4)). *Vegetation canopy coverage* (BVC) was calculated by Equation (5) ^27^.

1 $$VPC=100 \% \times \frac{\sum _{i=1}^{6}Aer{a}_{i}}{Are{a}_{BEU}}\,\,i=1,2,\ldots \ldots ,6$$

Where, *Area*_*BEU*_ is the area of a BEU; *Area*_*i*_ is the area of each vegetation type in the BEU; *i* is the vegetation types, including arboreal forest land, open forest land and shrub land, high-, mid- and low-cover grassland.

2 $$RD=100 \% \times \frac{\sum _{i=1}^{5}Aer{a}_{i}}{Are{a}_{BEU}}\, i=1,2,\ldots \ldots ,5$$

Where, *Area*_*i*_ is the area of each kind of human activity in the BEU; *i* is the human activities, including farmland, urban, build-up land, rural resident land and pasture.

3 $${\rm{Range}}\,{\rm{standardise}}\,(X)=100\times \frac{X-{X}_{min}}{{X}_{max}-{X}_{min}}$$

4 $$NWC=100 \% \times (\sum _{i=1}^{Ns}Are{a}_{C}/\sum _{i=1}^{Ns}Are{a}_{R})$$

Where, *Area* is the area of natural wetland with closely hydraulic connection with river; *C* is the year of evaluating PSI, and *R* is the historical year; *Ns* is the number of wetland.

5 $$BVC=100\times [(NDVI-NDV{I}_{soil})/(NDV{I}_{veg}-NDV{I}_{soil})]$$

Where, Normalized Difference Vegetation Index (NDVI) was calculated using the September OLI images; *NDVI*_*soil*_ and *NDVI*_*veg*_ represent the NDVI in the vegetated and non-vegetated area, respectively. In this study, *NDVI*_*soil*_ was the NDVI of 5% cumulative frequency, and *NDVI*_*veg*_ was the NDVI of 95% cumulative frequency in the study area^28, 29^.

**Table 4:** The evaluating indicators and weights of PSI based on remote sensing method.

Indicators		Sub-indicators
Riparian Stability (RST) (0.5)	Bank stability (BKS) (0.25)	Bank slope (BS) (0.25)
		Water level width(WL)(0.25)
		Vegetation percent cover (VPC) (0.25)
		Riparian zone area (RA) (0.25)
	Vegetation coverage (BVC) (0.5)	Percent canopy cover
	Human disturbance (RD) (0.25)	Build up and farmland percent cover
River connectivity (RC) (0.25)		Human-built instream structures, including bridges, dams, culverts
Natural wetland conservation (NWC) (0.25)		Wetland area in the evaluating year (A_C_) and historical year (1976)(A_R_)

### Calculated PSI of the riparian zone

Sub-indicators and indicators of PSI based on remote sensing method were scored by modification of the score sheets of Chinese Ministry of Water Resources technical protocol (Table A, Supplement Materials). Each indicator was given a score between 0 and 100, with higher number implying better condition. The indicators were calculated with associated sub-indicators that were scored multiplying by their corresponding weights. The indicator of bank stability was calculated by the Equation (6). The PSI of riparian zone was calculated by the Equation (7).

6 $$BKS=(BS+WL+VPC+RA)/4$$

7 $$PSI=RST\times 1/2+RC\times 1/4+NWC\times 1/4\,or\,PSI=RST\times 2/3+RC\times 1/3$$

### Statistical validation of evaluation results

To validate evaluation results, an independent samples *t*-test and *F*-test were performed to examine the statistically significant difference in PSI scores between field-evaluation and remote sensing observation. A test of normality was first performed using the Kolmogorov-Smirnov statistic and Shapiro-Wilk statistic to examine the possibility that the PSI scores derived from 45 measurement sites conform to a normal distribution.

Under the circumstance of no significant difference, the study sought to understand the similarity between the remote sensing and field derived PSI, which led to the use of coefficients used for understanding model outputs. We used the common hydrological modeling coefficient of Nashe and Sutcliffe (NS) to evaluate similarity of riparian PSI between the field and remote sensing observation. NS is a method for quantitatively analyzing modeled outputs as they compare to observed values^30, 31^. The method was appropriate as we compared the observed field data and the modeled remote sensing approach.

### Spatial statistic and analysis of riparian PSI

The Getis-Ord General *Gi* and the Anselin local Moran’s *I* statistics were calculated to analyze the spatial distribution of riparian PSI. The General *Gi* indicator was used to identify concentrations of high or low PSI values, while Moran’s *I* indicator was applied for calculating spatial autocorrelation of PSI between each BEU and its neighboring BEUs, and identifying spatial outliers of PSI value^32–34^. A search radius or threshold distance is essential for the two local statistics. In this study, the ‘Incremental Spatial Autocorrelation’ Toolbox in ArcGIS was used to determine the optimal distance radius for spatial statistics analysis. This tool measures spatial autocorrelation for a series of distances and optionally creates a line graph of those distances and their corresponding *z*-score. The statistically significant peak *z*-scores indicate distance where spatial processes promoting clustering are most pronounced. The distance corresponding to the first peak *z*-score was adopted for spatial statistics analysis by the default recommendation.

The Getis-Ord General *G* statistic was calculated using the fixed Euclidean distance method with an optimal threshold distance by the ‘Hot-Spot Analysis’ Toolbox in ArcGIS. The Anselin local Moran’s *I* was calculated using the inverse distance method with the ‘Cluster and Outlier Analysis’ Toolbox. The existence of a dispersed or a clustered distribution of riparian PSI based on a *z*-score (the *standard deviation* about the *mean*) where the *p*-value of statistical significance is typically set at 0.05 or 0.01 (a 95% or 99% confidence interval (CI)). Positive *z*-scores indicated clustering of large PSI values and negative *z*-scores indicated clustering of small PSI values (e.g., a *z*-score of 2.58 indicated at the 99% CI BEUs with large neighbors, and −2.58 indicated BEUs with small neighbors).

## Results

### Riparian PSI

**Figure 5:**
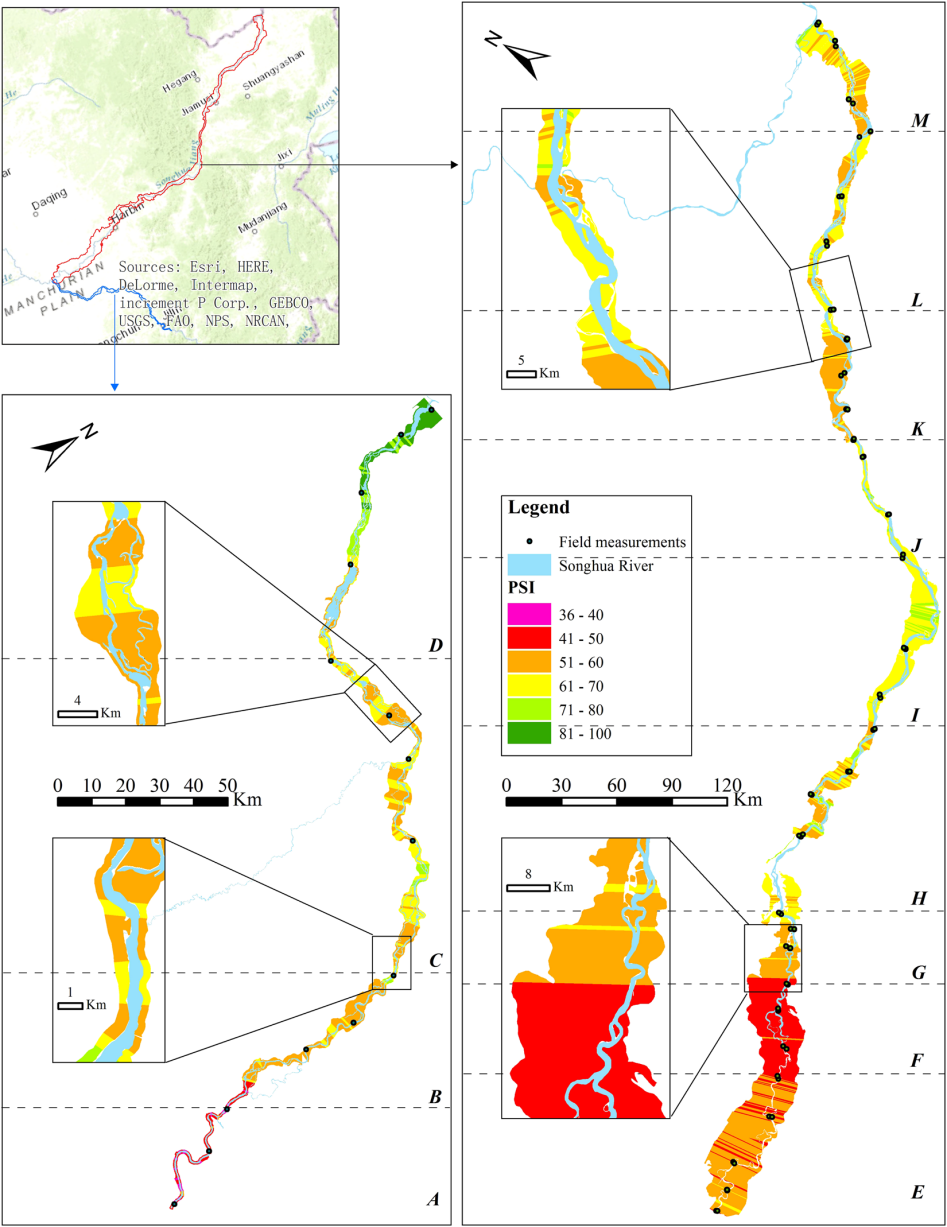
The spatial distribution of PSI values were derived from 2,081 BEUs. A-M represent the thirteen measurement sections. The map was prepared using ArcGIS 10.3 software (http://www.esri.com/software/arcgis). The map is credited to: Esri, HERE, DeLorme, Intermap, increment P Corp., GEBCO, USGS, FAO, NPS, NRCAN, GeoBase, IGN, Kadaster NL, Ordnance Survey, Esri Japan, METI, Esri China (Hong Kong), swisstopo, MapmyIndia, © OpenStreetMap contributors, and the GIS User Community.

The BEUs-based PSI results of the entire riparian zone were illustrated in Fig. 5. The *mean* and *standard deviation* of PSI, and number of BEUs for each measurement section were summarized in Table 5. The riparian condition had a significant difference among 13 measurement sections due to the range of PSI values. The dominated land cover types of riparian zones in section *A* and *F* is build-up category, and the riparian zones lost its ecological functions, resulted in PSI values below 45, which was lower than other measurement sections. In addition, the two sections with the smallest *standard deviation* (i.e., less than 2) indicated that the riparian zones were completely developed by human induced build-up structures. Section *D* has been less disturbed by human activities, and resulted in a better riparian condition, with the highest PSI value of over 70. However, the section also possessed the highest *standard deviation* up to 10.62, indicating a wide difference within the section. The PSI in section *I* and *J* was over 65 and the *standard deviation* was less than 3, indicating that the riparian zones have been in a homogeneous and stable condition. Section *B* with the 56.74 PSI value but a high standard deviation (5.20), indicating a weak stability of the riparian zone.

**Table 5:** The Mean, standard deviation of riparian PSI and number of BEUs in each measurement sections.

Measurement sections	*Mean*	*Standard deviation*	Number of BEUs
*A*	41.29	1.78	83
*B*	56.74	5.20	108
*C*	60.85	4.65	187
*D*	74.27	10.62	141
*E*	52.11	2.68	212
*F*	44.33	1.70	139
*G*	59.41	2.80	101
*H*	63.49	5.93	338
*I*	66.31	2.51	254
*J*	65.95	2.69	114
*K*	57.67	2.77	101
*L*	62.99	4.42	189
*M*	60.76	3.06	116

### Accuracy validation

**Figure 6:**
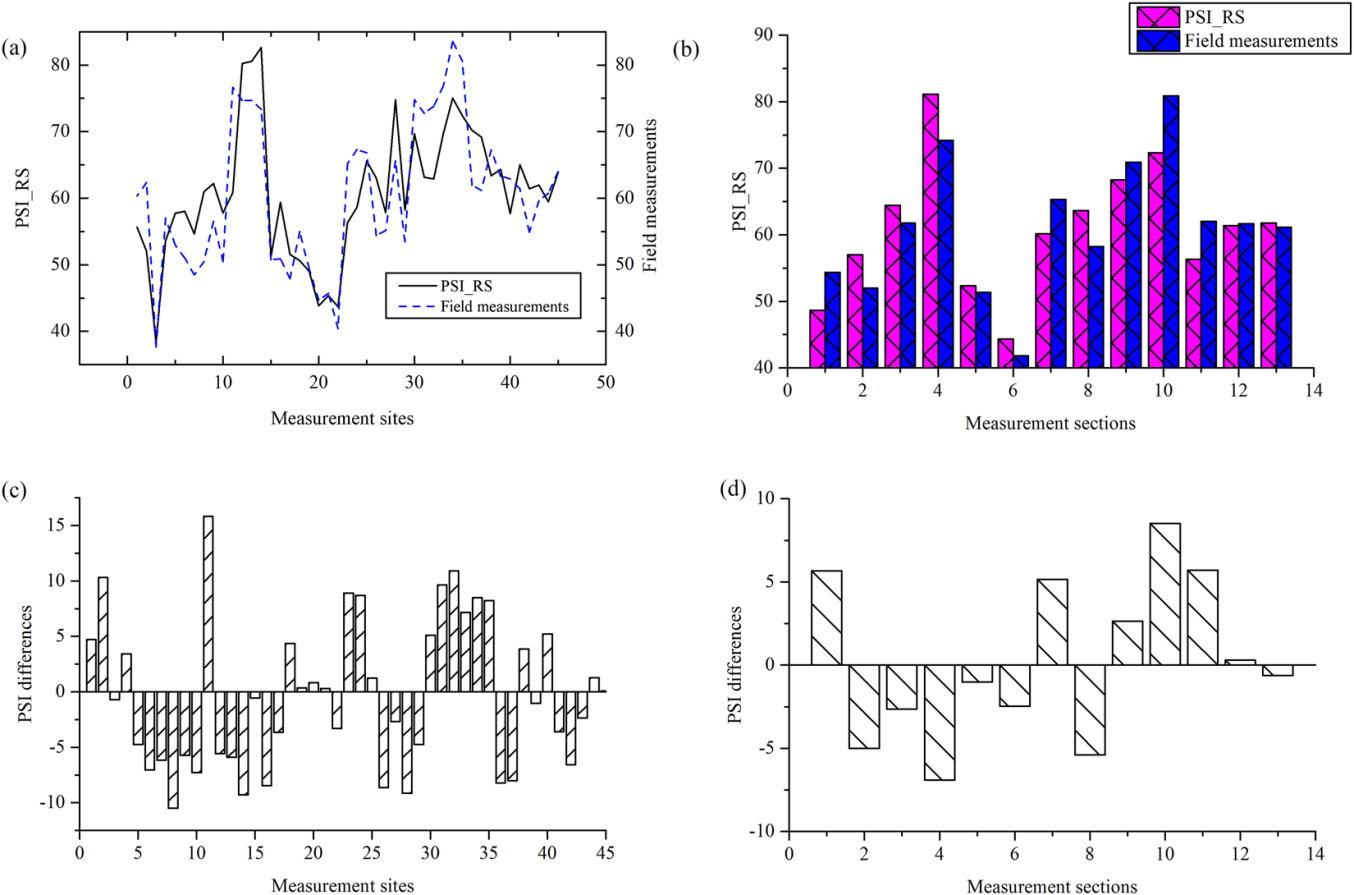
The evaluation results was validated by field measurements: (**a**) the comparison of 45 measurement sites; (**b**) the comparison of 13 measurement sections; (**c**) and (**d**) the differences of PSI values derived from corresponding to measurement sites and measurement sections.

The comparison between PSI derived from field measurements and remote sensing observation was implemented both in measurement sites and measurement sections to validate the evaluating results (Fig. 6). The trend of the riparian PSI was consistent in both measurement sites and measurement sections, and the riparian PSI values were between 38 and 85. Specifically, there were a little differences in some measurement sites and sections. The PSI differences between field measurements and remote sensing observations for all sites were mainly distributed between 0–10, with the exception of the eleventh measurement site, which produced the highest difference (15.84). Measurement sites with difference less than 5 account for over 44% of all measurement sites. The PSI differences between field measurements and remote sensing observations were less than 8 for all measurement sections. The measurement sections with the difference less than 3 account for over 46.2%. The tenth measurement section produced the highest difference of 8.52.

The normality test indicated that the PSI scores derived from 45 measurement sites represented a normal distribution (Shapiro-Wilk statistic, *p* = 0.51). The field and remote sensing derived measurements were compared with Students t-test and found that there was no significant difference between the two measurements (*p* = 0.16) (Table 6). Spatial statistic and analysis of riparian PSI

**Table 6:** The independent-samples *t*-test for field and remote sensing derived measurements.

	Levene’s Test for Equality of Variances		t-test for Equality of Means						
	F	Sig.	t	df	Sig. (2-tailed)	Mean Difference	Std. Error Difference	95% Confidence Interval of the Difference	
								Lower	Upper
Equal variances assumed	1.65	0.20	0.16	88	0.87	0.36	2.20	−4.02	4.74
Equal variances not assumed			0.16	86.51	0.87	0.36	2.20	−4.02	4.74

**Figure 7:**
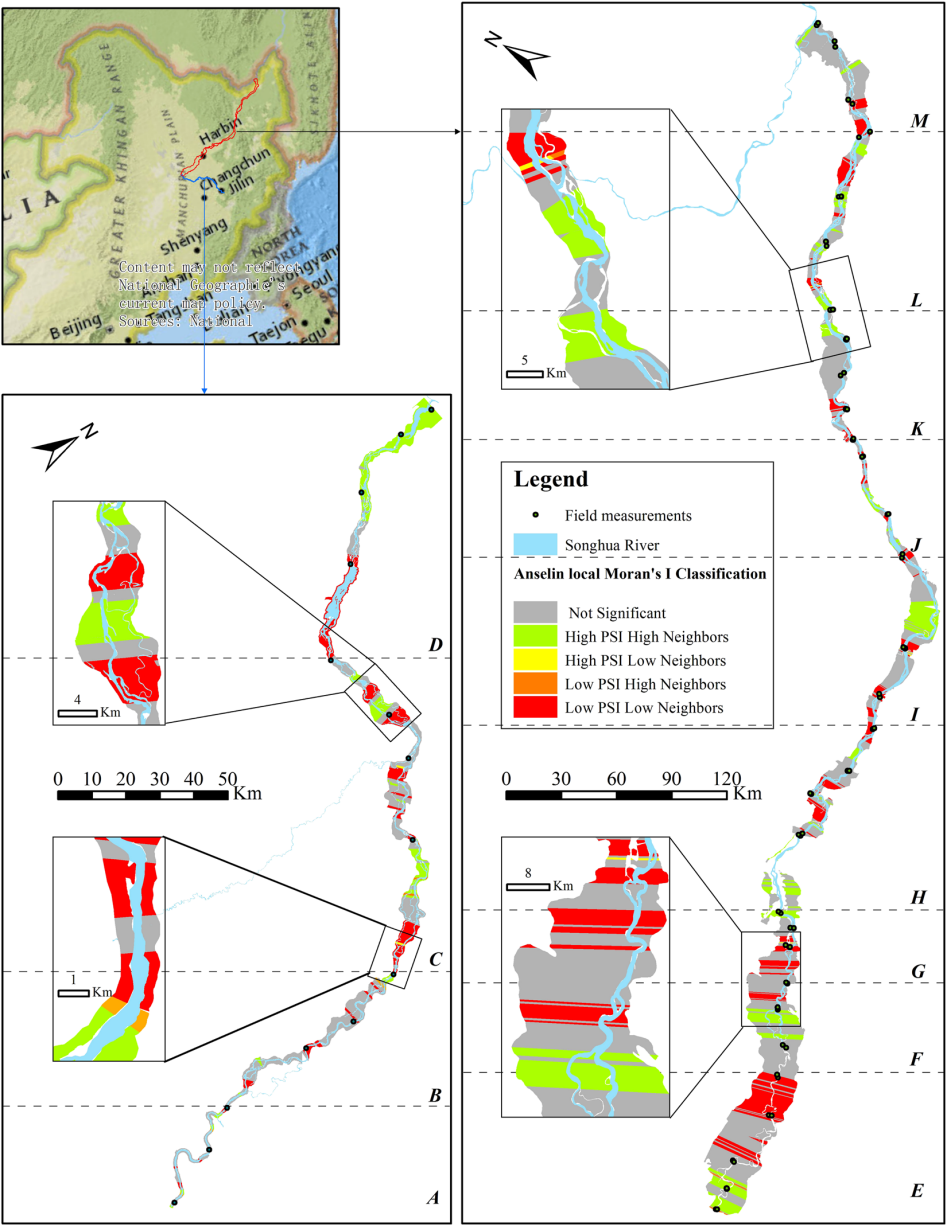
Anselin local Moran’s I statistics for the riparian PSI values was calculated and mapped by ArcGIS 10.3 software (http://www.esri.com/software/arcgis). A-M were the thirteen measurement sections. “High PSI High Neighbors” is that the riparian zone with a high PSI value is surrounded by the neighbors with high PSI values; “High PSI Low neighbors” is that the riparian zone with a high PSI value is surrounded by the neighbors with low PSI values; “Low PSI Low Neighbors is that the riparian zone with a low PSI value is surrounded by the neighbors with low PSI values. A-M were the thirteen measurement sections. The locus map is the World Topographic Map basemap within ArcGIS 10.3 software. The map is credited to: Esri, HERE, DeLorme, Intermap, increment P Corp., GEBCO, USGS, FAO, NPS, NRCAN, GeoBase, IGN, Kadaster NL, Ordnance Survey, Esri Japan, METI, Esri China (Hong Kong), swisstopo, MapmyIndia, © OpenStreetMap contributors, and the GIS User Community.

**Figure 8:**
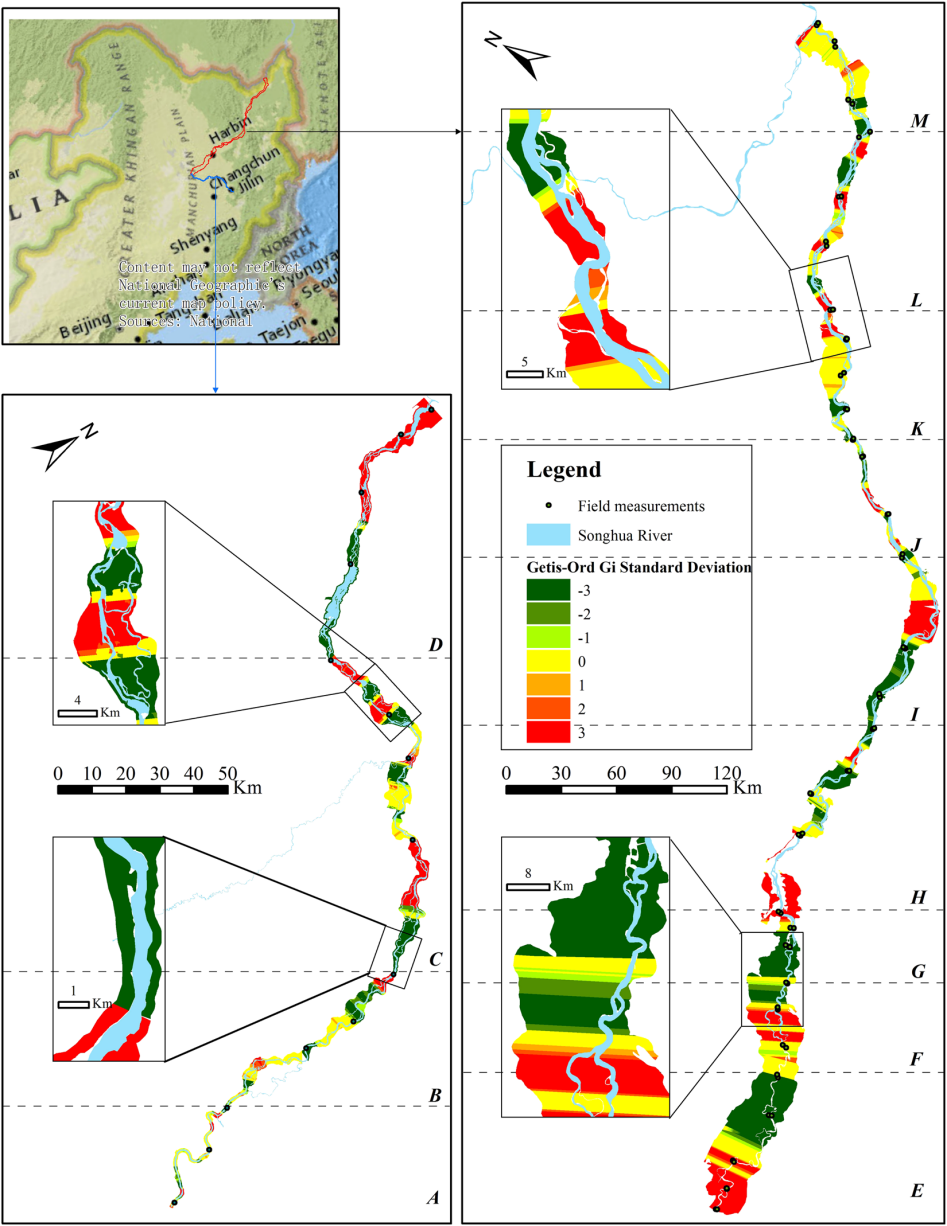
Getis-Ord Gi spatial statistics for the riparian PSI value was calculated and mapped by ArcGIS 10.3 software (http://www.esri.com/software/arcgis). Negative z-scores (z < −1.65, p < 0.1) represent statistically significant clusters of small PSI values, and become cold spots at the 90–95% confidence interval. Positive z-scores (z > 1.65, p < 0.1) indicates statistically significant clusters of high PSI values, and become hot spots at the 90–95% confidence interval. Clusters were non-significant (−1.65 < z < 1.65) in neighborhoods with a wide variation in PSI values, indicating those riparian conditions were randomly distributed. A-M were the thirteen measurement sections. The locus map is the World Topographic Map basemap within ArcGIS 10.3 software. The map is credited to: Esri, HERE, DeLorme, Intermap, increment P Corp., GEBCO, USGS, FAO, NPS, NRCAN, GeoBase, IGN, Kadaster NL, Ordnance Survey, Esri Japan, METI, Esri China (Hong Kong), swisstopo, MapmyIndia, © OpenStreetMap contributors, and the GIS User Community.

The Anselin local Moran’s *I* and Getis-Ord General *Gi* statistics were calculated to discriminate structural difference and look for evidence of clustering throughout the riparian zone (Figs 7 and 8). Anselin local Moran’s *I* statistic found that the high- high clusters, i.e., high PSI values with high neighbors, appeared in section *C*, *D*, *F*, *I* and *L*. However, the PSI value in the high cluster of section *F* was below 50, which was significant less than other sections. Section *A* and *B* with dominated urban and farmland area had the lowest PSI values, and did not present strong high-high clusters. The low-low clusters, i.e., low PSI values with low neighbors, presented in all measurement sections. A few cases presented outliers, in the form of low-high or high-low clusters for each sections. Within the section *D*, the riparian PSI value changed from low-low clusters to high-high clusters.

For the Getis-Ord *Gi* statistic, Negative z-scores (z < −1.65, p < 0.1) represent statistically significant clusters of low-low PSI values, and become cold spots at the 90–95% confidence interval. Positive z-scores (z > 1.65, p < 0.1) indicates statistically significant clusters of high-high PSI values, and become hot spots at the 90–95% confidence interval. Clusters were non-significant (−1.65 < z < 1.65) in neighborhoods with a wide variation in PSI values, indicating those riparian conditions were randomly distributed.

### Land use patterns in riparian zone

According to the evaluation results of the riparian PSI and its spatial statistics, the land use pattern was analyzed and found that the riparian landscape in the study area experienced a significant change from 1976 to 2013 (Tables 7 and 8). The areas of build-up and farmland categories accounted for the total section had increased in every measurement section from 1976 to 2013. In addition, the average annual change rate of build-up and farmland in each section had a positive growth, with the exception of section *K*, which the average annual change rate was −1.4‰ per year. The combination of riparian PSI values in each measurement section found that for section *A* and *F* with low PSI values, the build-up and farmland categories comprised over 80% of the total area from 1976 to 2013. However, in the section *D* with the highest PSI value, forest, grassland and wetland accounted for over 60% of the total area, especially over 80% from 1976 to 1986. For section *H*, *I*, *J* and *L* with the moderate PSI values, forest, grassland and wetland comprised over 45% of the total area. The average annual change rate of build-up and farmland of section *C*, *J* and *M* was over 14‰, more than other sections, which indicated that these sections were seriously disturbed during the 37 years. Section *A* and *F* with the lowest average annual change rate of build-up and farmland indicated that those riparian zones were changed into the urban and farmland area.

**Table 7:** The main land-use types comprise percent of the total area of the section in the riparian zone from 1976 to 2013. “*a*”: build-up and farmland; “*b*”: forest and grassland; “*c*”: wetland.

	1976			1986			1995			2000			2005			2013		
	Percentage (%)			Percentage (%)			Percentage (%)			Percentage (%)			Percentage (%)			Percentage (%)		
	*a*	*b*	*c*	*a*	*b*	*c*	*a*	*b*	*c*	*a*	*b*	*c*	*a*	*b*	*c*	*a*	*b*	*c*
*A*	83.7	11.4	5.0	80.5	14.9	4.6	86.5	7.8	5.7	87.3	6.8	5.9	86.9	8.2	4.9	89.4	9.4	1.2
*B*	86.0	10.4	3.6	81.1	16.8	2.1	84.4	7.0	8.6	87.4	8.6	4.1	81.7	12.4	5.9	92.9	3.6	3.5
*C*	33.8	31.8	33.5	44.9	44.8	5.4	29.7	37.7	31.3	34.3	46.7	17.7	43.2	35.5	20.6	58.5	29.4	11.4
*D*	25.7	21.9	51.6	14.4	53.2	27.9	14.8	37.1	48.0	15.7	38.4	45.6	25.0	32.8	41.9	35.8	33.6	30.3
*E*	45.8	10.4	42.5	44.4	8.5	44.7	56.9	6.2	3.3	46.9	16.2	34.5	47.8	20.0	29.8	57.5	16.8	23.2
*F*	88.0	1.8	10.0	88.1	3.1	8.5	90.0	1.9	7.9	89.3	1.7	8.8	89.1	1.8	8.9	90.7	1.7	7.3
*G*	51.7	22.0	26.2	48.1	2.7	49.2	53.6	40.3	6.1	50.9	39.5	9.6	51.0	30.5	18.3	55.8	29.3	14.8
*H*	33.1	24.4	42.5	39.3	15.7	45.0	37.9	26.1	36.0	36.7	22.9	40.4	40.8	19.0	40.2	42.5	18.3	39.2
*I*	43.7	6.9	49.4	43.5	5.7	50.8	44.2	5.5	50.3	49.3	5.6	45.1	49.8	5.6	44.5	51.7	4.7	43.8
*J*	6.6	28.3	65.0	5.2	25.6	69.2	8.8	62.3	28.9	8.0	37.5	54.5	25.0	34.3	40.7	43.4	33.3	23.3
*K*	72.8	2.6	24.6	72.0	5.9	22.0	75.1	18.0	6.8	65.5	1.8	32.7	66.8	1.9	31.3	69.1	1.9	28.7
*L*	47.2	7.9	43.3	56.5	8.5	35.0	60.6	6.8	32.2	50.9	20.2	27.4	50.9	20.9	26.7	52.6	20.1	25.7
*M*	26.7	56.7	16.6	37.5	3.7	58.8	44.3	3.5	52.3	60.7	22.7	16.6	60.9	22.1	17.0	62.1	21.8	16.1

**Table 8:** The average annual change rate of build-up and farmland in each section.

Sections	Average annual change rate of build-up and farmland (‰)					
	1976–1986	1986–1995	1995–2000	2000–2005	2005–2013	1976–2013
*A*	1.1	3.0	3.4	−5.8	5.4	1.9
*B*	−5.1	4.1	7.7	−14.6	15.8	2.2
*C*	24.8	−37.3	32.0	41.9	40.6	14.9
*D*	−61.5	4.7	16.5	88.8	20.9	2.3
*E*	−5.2	24.1	−32.3	6.4	20.1	5.8
*F*	−2.9	1.9	4.6	0.8	2.0	0.9
*G*	−8.0	12.1	−11.5	2.0	10.3	2.0
*H*	15.9	−2.8	−4.7	22.9	4.1	6.9
*I*	−3.8	1.7	27.6	3.4	3.6	4.4
*J*	−34.5	50.0	10.1	46.2	16.3	14.8
*K*	−4.2	3.9	−20.7	5.6	3.2	−1.4
*L*	18.3	7.5	−35.5	1.5	3.2	2.9
*M*	31.7	18.2	73.1	1.3	1.7	23.6

In addition, quantification of land use pattern of the BEUs in the high-high (hot spots) and low-low (cold spots) clusters found that build-up and farmland areas in sections *A*, *B* and *F* both accounted for over 70% of the total area in the hot spots and cold spots, while wetland, forest and grassland of the section *D* comprised over 70% of the total area (Table 9). In BEUs of the hot spots, wetland of section *E*, *H* and *J* accounted for over 47% of the total area, and forest and grassland of section *C* also accounted for over 47%. Build-up and farmland of section *G*, *I* and *K* comprised over 50% of the total area. In BEUs of the cold spots, build-up and farmland accounted for over 50% of the total area in all sections, with the exception of section *D*, *E*, *H*, *I* and *J*. Wetland, forest and grassland of section *H*, *I* and *J* accounted for over 50% of the total area.

**Table 9:** The main land-use types comprise percent of the total area of the BEUs in the hot spots and cold spots. “*a*”: build-up and farmland; “*b*”: forest and grassland; “*c*”: wetland.

Sections	Hot spots			Cold spots		
	*a*	*b*	*c*	*a*	*b*	*c*
*A*	73.20	16.52	4.75	93.17	2.72	4.11
*B*	84.59	14.14	1.27	95.69	1.81	2.50
*C*	27.09	47.15	24.69	56.51	27.78	15.02
*D*	23.09	26.93	49.98	19.32	50.83	23.84
*E*	47.45	2.52	47.68	48.34	33.39	13.03
*F*	89.35	1.51	7.11	93.85	2.67	3.15
*G*	69.82	7.25	21.89	59.33	30.85	7.17
*H*	15.43	21.53	62.74	45.26	7.69	46.73
*I*	61.61	3.83	27.43	36.14	3.12	60.38
*J*	10.19	22.80	65.85	9.66	48.19	40.79
*K*	50.08	1.48	47.34	71.88	1.71	23.72
*L*	46.20	17.56	30.19	50.68	21.08	24.82
*M*	38.87	36.38	15.18	54.48	19.53	16.39

## Discussion

The multi-metric method derived a single indicator of riparian PSI from the combination of a number of sub-indicators to evaluate riparian condition. The consistency of BEUs-based evaluation results with field measurements was validated by statistical *t*-test and the NS model coefficient. No significant difference between two evaluation results and the NS value of 0.63 demonstrated that remote sensing derived riparian PSI and evaluation of riparian condition was efficient, reliable and comparable. The BEUs-based method with sub-indicators calculated from remote sensing data was able to ensure objectiveness and reliability of evaluation results through reducing the concerns of accessibility in field-based investigation. As anthropogenic and natural factors drive constant change, repeated measurements over time is essential for monitor and manage the riparian zone. In this study, remote-sensing based method was able to monitor and evaluate the riparian condition repeatedly over time. BEUs-based evaluation results were able to provide a guideline for field measurements to select the typical and suitable measurement sites, and riparian protection. The BEUs-based evaluation approach developed from this representative plain river of the northeast China can be extend to the other plain rivers. As for mountainous rivers, this study provided a generalized approach for combining the indicators and sub-indicators to derive the PSI values. Modifications of scoring criteria and weights of sub-indicators and addition of sub-indicators may be needed for specific regions.

The PSI value is able to indicate riparian condition. A high PSI score indicated the land-use pattern was appropriate, and riparian ecosystem was in healthy condition. But it is necessary to consider the sub-indicators when interpreting the final PSI sores. That is because although two sites or BEUs may have the same PSI score, they may have very different sub-indicator scores for indicating environment problems. For example, low PSI score for sections *A* and *F* indicated that those sections of the frail riparian ecosystem were undermined, but did not provide information regarding specific problems. After analysis of sub-indicators scores, the high score of sub-indicator of *human disturbance* revealed that these section of riparian zone were disturbed by the land-use pattern. In those sections, the build-up and farmland accounted for over 80% of the total area. The forest land and wetland only made up 4–7.08%. Comparatively, sections *D*, *I* and *J* with high PSI scores, the wetland and forest covered over 50–60% of the total riparian zone, and farmland comprised about 30–40% of the landscape. These proportions indicated that it was essential to find a balance between riparian development and fluvial ecosystem preservation.

Field measurements were designed for site-scale assessments of the current condition of a riparian zone. With the limitation of the number of measurement sites, field-based method should be more appropriate to provide section-scale evaluation results. This study provided the BEUs-based evaluation results of riparian condition. The spatial statistics revealed variation of riparian PSI. Anselin local Moran’s *I* and Getis-Ord *Gi* statistics both can identify different kinds of PSI assemblages. The low-low clusters of riparian PSI discriminated the vulnerable area of the riparian zone. The information was essential for riparian protection to identify current or potential threats to an area, and adopt corresponding protective measures. Meanwhile, the high-high clusters were equally important for riparian management to identify the parts of riparian zone with a high PSI value in each section. However, the clusters were local comparisons of PSI values. It did not indicate the BEUs with the high-high clusters had a good riparian condition. For example, section *A* and *F* both had a high-high clusters of PSI values, but analysis of respective evaluation results derived from BEUs found that the PSI values were below 50 in all BEUs. The sections of riparian zone were disturbed by human activities, and in a poor condition. This demonstrated that the combination of spatial cluster analysis and BEU-based evaluation results was able to make a correct judgment for riparian condition.

Analysis of land use pattern of the BEUs in the cold and hot spots found that the sections with dominated urban and rural area have a low PSI value due to disturbance by human activities. The sections dominated wetland and forest possessed a high PSI values. The conclusions were consistent with reported studies that land-use types and patterns in a riparian zone have a great influence on riparian condition^13, 35–39^. Analysis of land use data from 1978 to 2013 found the riparian landscape has a great change. The build-up and farmland categories accounted for over 35% of the total area in each section, more than other land cover types indicated that build-up and farmland have been the main human disturbance to the riparian zone. In addition, the positive average annual change rate of build-up and farmland indicated that the riparian zone had been developed by the human activities from 1976 to 2013. An appropriate land use plan is needed with emphasis on protecting the riparian ecosystem and maintaining a balance between economic development and ecosystem health in the future.

Analysis of the sub-indicator scores of *bank stability* found that section *C, D, L and M* with the highest PSI values possessed the highest scores of over 60. But section *A*, *F* and *G* with the lower PSI values processed higher scores (about 50) of *bank stability* than sections *B*, *I* and *J* (less than 50). This indicated that the trend of the riparian PSI values was inconsistent with the scores of *bank stability*. This difference revealed that river connectivity and riparian vegetation had a great influence on riparian condition. River banks in the section *A*, *F* and *G* had realigned or modified by human-built structures or activities (e.g., bridges, weirs, dam, walking tracks, hard footpaths and recreation access), which created hard concrete banks and weakened bank erosion, resulted in high scores. Section *B*, *I* and *J* in the rural area could be influenced by agricultural practices or overgrazing, and caused soils degradation and accelerated bank erosion. While human-built structures precluded the lateral connectivity between the river channel and its floodplain, changed the longitudinal continuity of natural riparian vegetation, and decreased riparian vegetation coverage. In this study, section *A*, and *F* with below 10% of vegetation cover and a number of weirs resulted with the low scores of *river connectivity* and *percent canopy cover*. These demonstrated that bank stability, river connectivity and vegetation structure are closely related with riparian quality.

## Conclusions

A BEUs method of remote sensing assessment was used to calculate the riparian PSI of Songhua River, Northeast China. No significant difference between evaluation results was found when compared with field measurements. The conclusions confirmed the evaluation result based on BEUs was accurate and remote sensing method was available to evaluate the riparian PSI. The NS model coefficient quantified the similarity of PSI values between the two approaches, which demonstrates that remote sensing derived PSI values are promising to facilitate repeated monitoring PSI of a riparian zone. Evaluation results derived from 13 measurement sections indicated that over 60% of riparian zone were disturbed by human activities, and the PSI values were below 65. Anselin local Moran’s *I* and Getis-Ord *Gi* statistics were able to identify the variation and clusters of riparian PSI. Land use patterns of riparian zone had an important effect on riparian condition. The dominated build-up and farmland area had a poor riparian condition and the dominated wetland and forest area had a good riparian condition. The build-up and farmland had been the main human disturbance to the riparian condition. In addition, the two land cover types increased over the past 37 years. The area of build-up and farmland had increased by 787.9 km^2^ from 3476.0 km^2^ in 1976 to 4263.9 km^2^ in 2013.

## Electronic supplementary material

Supplementary Information
