# Supplementary material for: Evaluation of riparian condition of Songhua River by integration of remote sensing and field measurements
Source: Sci Rep. 2017 May 31;7:2565. doi: 10.1038/s41598-017-02772-3 (PMC5451399; doi:10.1038/s41598-017-02772-3)
Supplement: Supplementary file 1 — Supplementary Information [file 41598_2017_2772_MOESM1_ESM.docx]

## Table A Supplement Materials for manuscript SREP-16-38374A

Evaluation of riparian condition of Songhua River by integration of remote sensing and field measurements

Table A. Score sheets: Sub-indicators and indicators of PSI based on remote sensing method were scored by reference and modification of the score sheets of Chinese Ministry of Water Resources technical protocol.

Table 1. The score sheet of bank stability

| Score | 25 | 50 | 75 | 90 |
| --- | --- | --- | --- | --- |
| Bank slope (°) | >60 | 30-45 | 15-30 | <15 |
| Water level width | <25 | 25-50 | 50-75 | >75 |
| Vegetation percent cover (%) | <25 | 25-50 | 50-75 | >75 |
| Riparian zone area | <25 | 25-50 | 50-75 | >75 |

Table 2. The score sheet of canopy cover

| % cover of forest, shrub and grass of any height | Score |
| --- | --- |
| 0 | 0 |
| 0-10% | 25 |
| 10%-40% | 50 |
| 40%-75% | 75 |
| >75% | 100 |

Table 3. The score sheet of human activity

| % cover of human activities^*^ in the BEUs | Score |
| --- | --- |
| 0 | 100 |
| 0-10% | 75 |
| 10%-40% | 50 |
| 40%-75% | 25 |
| >75% | 0 |

*Human activities including build up and farmland

Table 4. The score sheet of River connectivity

| Human-built instream structures within measurement section(e.g. bridges, culverts, weirs, dams) | Score |
| --- | --- |
| >4 instream structures | 0 |
| 3 instream structures | 25 |
| 2 instream structures | 50 |
| 1 instream structures | 75 |
| No instream structures | 100 |

Table 5. The score sheet of natural wetland conservation

| Wetland area ratio of evaluating year and historical year | Score |
| --- | --- |
| 86%-93% | 100 |
| 73%-86% | 75 |
| 45%-72 | 50 |
| 17%-44% | 25 |
| 0-16% | 0 |
